# Association of RNA m^7^G Modification Gene Polymorphisms with Pediatric Glioma Risk

**DOI:** 10.1155/2023/3678327

**Published:** 2023-01-24

**Authors:** Jinhong Zhu, Xiaoping Liu, Wei Chen, Yuxiang Liao, Jiabin Liu, Li Yuan, Jichen Ruan, Jing He

**Affiliations:** ^1^Department of Pediatric Surgery, Guangzhou Institute of Pediatrics, Guangdong Provincial Key Laboratory of Research in Structural Birth Defect Disease, Guangzhou Women and Children's Medical Center, Guangzhou Medical University, Guangdong Provincial Clinical Research Center for Child Health, Guangzhou, 510623 Guangdong, China; ^2^Department of Clinical Laboratory, Biobank, Harbin Medical University Cancer Hospital, Harbin, 150040 Heilongjiang, China; ^3^Department of Hematology, Guangzhou Women and Children's Medical Center, Guangzhou Medical University, Guangzhou, 510623 Guangdong, China; ^4^Department of Neurosurgery, Xiangya Hospital, Central South University, Changsha, 410008 Hunan, China; ^5^Department of Pathology, Guangzhou Women and Children's Medical Center, Guangzhou Medical University, Guangzhou, 510623 Guangdong, China; ^6^Department of Hematology, The Second Affiliated Hospital and Yuying Children's Hospital of Wenzhou Medical University, Wenzhou, 325027 Zhejiang, China

## Abstract

Glioma stemming from glial cells of the central nervous system (CNS) is one of the leading causes of cancer death in childhood. The genetic predisposition of glioma is not fully understood. METTL1-WDR4 methyltransferase complex is implicated in tumorigenesis by catalyzing N7-methylguanosine (m^7^G) modification of RNA. This study is aimed at determining the association of glioma risk with three polymorphisms (rs2291617, rs10877013, and rs10877012) in *METTL1* and five polymorphisms (rs2156315 rs2156316, rs6586250, rs15736, and rs2248490) in *WDR4* gene in children of Chinese Han. We enrolled 314 cases and 380 controls from three independent hospitals. Genotypes of these polymorphisms were determined using the TaqMan assay. We found the *WDR4* gene rs15736 was significantly associated with reduced glioma risk (GA/AA vs. GG: adjusted odds ratio = 0.63, 95%confidence interval = 0.42 − 0.94, *P* = 0.023) out of the eight studied polymorphisms. Stratified analyses showed that the association of rs15736 with the risk of glioma remained significant in children aged 60 months or older, girls, the subgroups with astrocytic tumors, or grade I + II glioma. We also found the combined effects of five *WDR4* gene polymorphisms on glioma risk. Finally, expression quantitative trait locus (eQTL) analyses elucidated that the rs15736 polymorphism was related to the expression level of *WDR4* and neighboring gene *cystathionine-beta-synthase* (*CBS*). Our finding provided evidence of a causal association between *WDR4* gene polymorphisms and glioma susceptibility in Chinese Han children.

## 1. Introduction

The annual incidence of primary brain tumors is 6.14 per 100,000 in children under 19 years of age, as declaimed by the Central Brain Tumor Registry of the United States (CBTRUS). Among them, 11.9% of cases are malignant glioma [1]. Gliomas arise from glial cells of the central nervous system (CNS), including astrocytes, oligodendrocytes, and ependymal cells. Low-grade gliomas (LGGs), classified as World Health Organization (WHO) grade I or II lesions of the CNS, are generally not lethal [2]. However, due to comorbidities caused by the tumors and/or clinical intervention and their inclination to multiply relapses, the leading brain tumors developed in childhood often demand decades of management [3]. In addition, pediatric high-grade gliomas (pHGGs), classified as grade III or IV lesions, constitute one-third of gliomas, accountable for the most cancer-related deaths in children younger than 19 years old [4–6].

Environmental factors, including ionizing radiation, some toxic agents (N-nitroso compounds and pesticides), air pollution, and radiofrequency electromagnetic waves, have been suspected to be potentially implicated in the carcinogenesis of brain tumors. However, only ionizing radiation is well-established as a risk factor for brain tumors [7]. On the other hand, heredity is shown to play a role in glioma susceptibility: (1) familial aggregation: individuals with a family history of glioma are at increased risk of developing these tumors. (2) Some well-recognized genetic syndromes confer glioma risk, including the Turcot and Li-Fraumeni syndromes, neurofibromatosis type 1, and multiple enchondromatosis [8]. Moreover, numerous candidate gene association studies provide evidence of a link between genetic variation and glioma predisposition [8–12]. For instance, glioma susceptibility loci have been comprehensively explored in DNA repair, cell cycle, metabolism, and inflammation (including allergies and infections) pathways [8]. Moreover, a dozen glioma susceptibility loci are discovered in *CCDC26*, *PHLDB1*, *TERT*, *RTEL1*, *TP53*, *EGFR*, and *CDKN2A-CDKN2B* genes, using genome-wide association studies (GWASs) [13–16]. Like other cancers, the inherited risk of glioma may be a consequence of the coinheritance of many low-penetrant and low-risk gene single nucleotide polymorphisms (SNPs). It is crucial to identify more risk determinants to understand variations in and refine the predicting capacity of glioma predisposition.

N7-methylguanosine (m^7^G) is one of the most predominant modifications occurring in mRNA, miRNA, and tRNA [17–20]. This type of chemical modification is installed by a specific methyltransferase complex composed of WD repeat domain 4 (WDR4) and methyltransferase-like 1 (METTL1) [21]. WDR4, the non-catalytic component, can stabilize and enhance the methyltransferase activity of METTL1 [21]. m^7^G modification is an essential posttranscriptional mechanism to regulate cell fate and growth. Defective m^7^G tRNA modification caused by *METTL1* knockout impaired the differentiation and growth of embryonic stem cells [22]. Mutation of *WDR4* is causative for a heterogeneous group of microcephalic primordial dwarfism and Galloway-Mowat syndrome [23, 24]. A few publications suggest that dysregulated m^7^G tRNA modification is also implicated in carcinogenesis, including lung cancer, esophageal squamous cell carcinoma, intrahepatic cholangiocarcinoma, head and neck squamous cell carcinoma, and colon cancer [25–30]. Nevertheless, the roles of RNA m^7^G modification have not been reported in glioma. Genome-wide annotation of genetic variation by the Human Genome Project enabled us to evaluate the association between the genetic variants in the *METTL1* and *WDR4* genes and glioma susceptibility in a three-center case-control study (314 cases vs. 380 controls) with Chinese children of Han ethnicity.

## 2. Methods and Materials

### 2.1. Study Subjects

Three medical centers participated in this study. Guangzhou Women and Children's Medical Center, Xiangya Hospital, The Second Affiliated Hospital and Yuying Children's Hospital of Wenzhou Medical University were located in Guangzhou, Changsha, and Wenzhou, respectively [31]. We enrolled 314 cases diagnosed and histopathologically confirmed with glioma regarding the 2016 World Health Organization classification of tumors of the CNS. Blood samples were collected from cases prior to radiotherapy or chemotherapy. A total of 380 cancer-free controls were recruited from the same participating hospitals during the same periods (Table S1). Patients and healthy controls were age and sex-matched. All participants were not genetically related, and their patients or guardians provided written informed consent before the study commencement. The study protocol was authorized by the Institutional Review Boards of the participating hospitals. This study was conducted in accordance with the Declaration of Helsinki.

### 2.2. SNP Selection and Genotyping

By combining the NCBI dbSNP database and SNPinfo online tool (https://snpinfo.niehs.nih.gov/), we choose three (rs2291617, rs10877013, and rs10877012) and five (rs2156315 rs2156316, rs6586250, rs15736, and rs2248490) potentially functional SNPs in *METTL1* and *WDR4* genes, respectively. Selection criteria were described previously [32, 33]. Genomic DNA was derived from peripheral blood samples of both cases and controls using Tiangen blood DNA extraction kits (Tiangen Biotechnology, Beijing, China). TaqMan genotyping method was adopted to evaluate the genotypes of samples for the selected SNPs with the probes were purchased from ABI (Applied Biosystems, Foster City, CA). All assays were run on an ABI 7900 (Applied Biosystems, Foster City, CA, USA) by the laboratory personnel blind to case/control status of samples. Both negative and positive were routinely included in each 384-well plate. Generally, 10% of samples were genotyped at random, and only a 100% concordance rate of the duplicated samples was accepted.

### 2.3. Statistical Analysis

The agreement of genotypes with Hardy-Weinberg equilibrium (HWE) was assessed by a goodness-of-fit *χ*^2^ test among control subjects. The demographic features were compared between the cases and controls using the *χ*^2^ test or *t*-test when appropriate. Multivariate logistic regression analyses were used to calculate the odds ratios (ORs) and 95% confidence intervals (CIs) after adjusting for age and sex. We also performed stratified analyses based on age, sex, subtypes, and tumor grade. Expression quantitative trait locus (eQTL) analyses of the significant SNPs were conducted using the Genotype-Tissue Expression (GTEx) (https://gtexportal.org). The *α* of 0.05 was used to define the statistical significance level. All statistical analyses were two-sided and carried out using the SAS v10.0 (SAS Institute, Cary, NC, USA).

## 3. Results

### 3.1. Association Study

Single SNP analyses indicated that only the *WDR4* gene rs15736 was significantly associated with glioma susceptibility (GA/AA vs. GG: adjusted OR = 0.63, 95%CI = 0.42 − 0.94, *P* = 0.023) out of the eight studied SNPs (Table 1). Children harboring minor allele rs15736 A were at a significantly reduced risk of developing glioma compared with those with two G alleles. More, *METTL1* gene rs10877012 was borderline significantly associated with increased glioma risk (adjusted OR = 1.45, 95%CI = 0.95 − 2.21, *P* = 0.086) under the recessive model. It was also the case for *METTL1* gene rs2291617 (adjusted OR = 1.42, 95%CI = 0.94 − 2.16, *P* = 0.098).

### 3.2. Stratified Analysis

Stratified analyses were performed by age, sex, glioma subtypes, and grades. Stratified analysis for *METTL1* gene SNPs demonstrated no significant association with glioma among any subtypes (Table 2). We also carried out stratified analysis for *WDR4* gene SNPs (Table 3) and found that rs15736 showed significant protective effects in children aged 60 months or older (adjusted OR = 0.58, 95%CI = 0.34 − 0.995, *P* = 0.048), girls (adjusted OR = 0.55, 95%CI = 0.31 − 0.98, P = 0.042), the subgroup with astrocytic tumors (adjusted OR = 0.54, 95%CI = 0.34 − 0.87, *P* = 0.011), and those with grade I + II glioma (adjusted OR = 0.58, 95%CI = 0.37 − 0.92, *P* = 0.019). We next conducted a stratified analysis for the combined effect of *WDR4* gene SNPs (Table 3). The concurrence of five indicated protective genotypes exhibited decreased glioma risk in kids presenting astrocytic tumors (adjusted OR = 0.60, 95%CI = 0.35 − 0.90, *P* = 0.016) and those with grade I + II glioma (adjusted OR = 0.60, 95%CI = 0.38 − 0.95, *P* = 0.029).

### 3.3. Expression Quantitative Trait Locus (eQTL) Analyses

**Figure 1:**
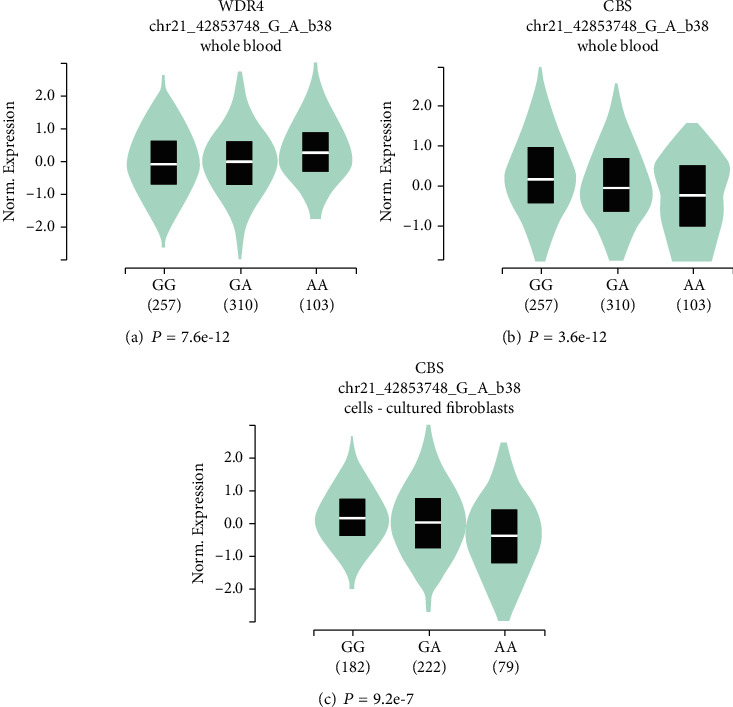
Expression quantitative trait loci (eQTL) analyses of *MDR4* gene rs15736 polymorphism using the public database GTEx portal. (a) The genotype of rs15736 and expression of *MDR4* gene in whole blood. (b) The genotype of rs15736 and expression of its nearby gene *CBS* in whole blood. (c) The genotype of rs15736 and expression of the *CBS* gene in cultured fibroblasts.

One of the crucial mechanisms underlying the association between causal SNPs and disease risk is affecting the expression levels of host genes. Given the significant association between rs15736 and the risk of glioma, we further assessed the effects of SNP on the expression of the target gene by taking advantage of released data from GTEx. In terms of whole blood, the expression levels of *WDR4* in subjects with AA genotype were significantly enhanced (Figure 1(a)), but the same individuals had the lowest levels of *cystathionine-beta-synthase* (*CBS*) (Figure 1(b)), an adjacent gene of *WDR4* among individuals with GG, GA, and AA genotypes. The data derived from cultured fibroblasts further verified the association of rs15736 AA genotypes with reduced *CBS* levels (Figure 1(c)). The cis-eQTL analysis indicates that rs15736 polymorphism may lead to altered expression of target genes.

## 4. Discussion

Pediatric glioma seriously threatens the life or impairs the quality of life of affected children. Apart from the environmental risk factors, genetic predisposition for glioma has been substantiated in mounting candidate gene-associated studies and GWASs [9]. Variations in genes involved in DNA repair, cell cycle, metabolism, and inflammation pathways have been recognized as a key basis of inherited glioma susceptibility, such as *PRKDC*, *XRCC1*, *PARP1*, *ERCC1*, *ERCC2*, *EGF*, and *IL13* [34]. Besides, polymorphisms of other important genes also contribute to glioma risk. For instance, a functional SNP rs12803321 with *Solute Carrier Family 25 Member 26* as the target gene was found to be a causal locus of LGG [12]. Recently, our group investigated the impacts of SNPs in the RNA m6A modification core genes on pediatric glioma susceptibility. We found that several SNPs were associated with the increased risk of glioma, including the *WTAP* rs7766006, *YTHDF2* rs3738067, and *FTO* rs9939609 polymorphisms [11]. Moreover, compared with adult glioma, genetic association studies are extremely few in pediatric glioma, and the sample sizes of studies are usually small because of the rarity of the disease [34].

In the present moderate-size case-control study, we found that the *WDR4* gene rs15736 was significantly associated with decreased glioma risk. Stratified analysis indicated that the protective effects of rs15736 remained prominent in several subgroups, including children aged 60 months or older, girls, astrocytic tumors, and grade I + II glioma. We also observed decreased glioma susceptibility for children with five protective genotypes in astrocytic tumors and grade I + II glioma subgroups. The findings suggest an association between the *WDR4* gene SNPs and pediatric glioma risk. At present, there are only two studies about genetic variations in the *METTL1*/*WDR4* gene and diseases. One study demonstrated that the *WDR4* gene rs465663 polymorphism might predispose to asthenozoospermia [35]. The other study, a GWAS performed by the Australian and New Zealand Multiple Sclerosis Genetics Consortium, identified the SNP (rs703842) positioned at the 3′ untranslated region (3′ UTR) of the *METTL1* gene as a multiple sclerosis susceptibility loci [36].

Over the past years, accumulating evidence is emerging to demonstrate the implication of the METTL1/WDR4 m^7^G methyltransferase complex in tumorigenesis. The upregulated expression levels of METTL1 and/or WDR4 and their association with prognosis were observed in multiple types of cancer, including lung cancer [29], esophageal squamous cell carcinoma (ESCC) [30], intrahepatic cholangiocarcinoma (ICC) [27], hepatocellular carcinoma (HCC) [37], and glioma [38]. Further investigation unveiled that METTL1/WDR4 accelerated lung cancer cell proliferation and migration by enhancing tRNA m^7^G modification and oncogenic mRNA translation, particularly CCND3 and CCNE1, the cell-cycle regulators [29]. WDR4 is a partner of METTL1, functioning by stabilizing and augmenting the methyltransferase activity of METTL1. Therefore, the low level or loss of function of WDR4 may comprise METTL1's methyltransferase activity to reduce m7G tRNA modification. Accumulating evidence has demonstrated the importance of WDR4 in carcinogenesis and drug resistance. Han et al. observed significantly overexpressed METTL1 and WDR4 in ESCC, which were associated with unfavorable outcomes in ESCC. The mechanistic study indicated that silencing *METTL1* or *WDR4* decreased m7G-modification levels of tRNAs, thereby downregulating the translation of many oncogenic genes in the RPTOR/ULK1/autophagy pathway [30]. An unbiased proteomic screening of differentially expressed genes between parental and lenvatinib-resistant HCC cells unveiled that METTL1 and WDR4 were highly elevated in drug-resistant cells. METTL1/WDR4-catalyzed m7G tRNA modification led to drug resistance by facilitating the translation of EGFR pathway genes. METTL1 depletion overcame resistance by inhibiting proliferation and inducing apoptosis of HCC cells [39]. In ICC, METTL1 and WDR4 enhanced the translation of cell-cycle and epidermal growth factor receptor (EGFR) pathway genes in an m^7^G-tRNA-dependent manner. On the contrary, some groups found that MTC may also be tumor-suppressing [19, 28]. Pandolfini et al.'s study indicated that the METTL1 increased tumor suppressor let-7 miRNA levels and thereby suppressed lung cancer cell migration [19]. Mechanistic investigation elucidated that besides translation regulation, METTL1 can also catalyze m^7^G modification of the primary miRNA transcript (pri-miRNA) to affect their processing. The m^7^G in precursors of let-7 miRNA interrupted inhibitory RNA secondary structures so as to expedite its maturation [19]. Another group reported that METTL1 inhibited colon cancer by increasing *let-7e* miRNA and decreasing its target gene *HMGA2*. Moreover, overexpression of METTL1 enhanced the cytotoxic effects of cisplatin on colon cancer cells by triggering the miR-149-3p/S100A4/p53 axis [28]. Thus far, only one publication showed the potential implication of m^7^G methyltransferase complex METTL1/WDR4 in glioma. Expression of METTL1 was augmented in glioma in comparison to normal tissue, and its expression levels were inversely associated with prognosis in glioma. In vitro experiments revealed that silencing of *METTL1* led to retardation of glioma cell growth [38]. However, several studies demonstrated the crucial role of m^7^G in neurodevelopment. MTC is in charge of installing the highly conserved m^7^G_46_ (7-methylguanosine) modification in tRNA. tRNA is well-known to facilitate protein synthesis by transporting amino acids to the expanding peptide chain during the corresponding mRNA translation. Intriguingly, the brain seems to be vulnerable to dysregulated tRNA modification. Recently, Shaheen et al. reported that mutation in *WDR4* disrupted tRNA m^7^G_46_ methylation, consequently leading to microcephalic primordial dwarfism [23]. Consistently, Braun et al. discovered a disease-causing mutation in the *WDR4* gene for Galloway-Mowat syndrome (GAMOS) characterized by neurodevelopmental defects [24]. Coincidentally, Lin et al.'s group demonstrated that the knockout of METTL1 in mouse embryonic stem cells (mESCs) preferentially affected the translation of cell cycle genes and genes associated with brain abnormalities. Moreover, self-renewal and neural differentiation of *METTL1* or *WDR4* knockout mESCs were severely impeded [40]. Overall, METTL1 may play context-dependent roles in tumorigenesis. In the current study, our results support METTL1 as a pediatric glioma susceptibility gene. The underpinning mechanism of how *METTL1* SNPs modify glioma susceptibility warrants further investigation.

Interestingly, we also found that *WDR4* SNP rs15736 was associated with an alteration in the expression of a neighboring gene *CBS*. CBS is an enzyme that generally forms a homotetramer to convert homocysteine to cystathionine, the first reaction in the transsulfuration pathway. CBS is dysregulated in different types of cancer, which is upregulated in kidney, colorectal, ovarian, lung, and breast cancer but downregulated in glioma and liver cancer. The low expression level of CBS is associated with poor survival in cancers with CBS as a tumor suppressor [41]. Zheng et al. reported that Apolipoprotein C1 could facilitate glioblastoma tumorigenesis by inhibiting CBS-mediated ferroptosis, suggesting that reduced expression may help to maintain glioblastoma cell survival by reducing ferroptosis [42]. These results suggest that affecting nearby genes is an alternative mechanism by which an SNP modifies disease susceptibility.

Several limitations of the study should be addressed. First, pediatric glioma is a rare disease. Although we recruited samples from three independent medical centers, the sample size of our cohort is still moderate. Second, gene-gene and gene-environment interactions were not taken into account. Third, functional experiments need to be performed to evaluate the effect of significant SNPs on gene expression. Finally, the effects of the SNP on the survival of glioma patients should be estimated. Unfortunately, we have no survival information on these glioma patients. Besides, no public database is available to evaluate the effect of polymorphisms on pediatric glioma patient survival by Kaplan-Meier analysis.

## 5. Conclusion

In conclusion, we identified glioma susceptibility loci in the *WDR4* gene for Chinese Han Children. Validation studies should be conducted in different populations.

## Figures and Tables

**Table 1:** Association of m^7^G modification genes and glioma risk in Chinese children.

Gene	Polymorphism	Allele		Case (*N* = 312)			Control (*N* = 380)			AOR (95% CI)^a^	*P* ^a^	AOR (95% CI)^b^	*P* ^b^	HWE
		A	B	AA	AB	BB	AA	AB	BB					
*METTL1*	rs2291617	G	T	127	129	56	155	175	50	1.00 (0.74-1.36)	0.984	1.42 (0.94-2.16)	0.098	0.956
*METTL1*	rs10877013	T	C	134	128	50	160	175	45	0.97 (0.71-1.31)	0.837	1.39 (0.90-2.15)	0.142	0.786
*METTL1*	rs10877012	T	G	129	128	55	152	180	48	0.95 (0.70-1.29)	0.742	1.45 (0.95-2.21)	0.086	0.639
*WDR4*	rs2156315	C	T	184	113	15	232	135	13	1.10 (0.81-1.50)	0.535	1.47 (0.69-3.14)	0.324	0.213
*WDR4*	rs2156316	C	G	146	144	22	163	186	31	0.86 (0.64-1.16)	0.323	0.85 (0.48-1.50)	0.563	0.027
*WDR4*	rs6586250	C	T	261	51	0	301	74	5	0.74 (0.50-1.09)	0.124	/	/	0.852
*WDR4*	rs15736	G	A	266	46	0	299	76	5	0.63 (0.42-0.94)	0.023	/	/	0.945
*WDR4*	rs2248490	C	G	148	143	21	165	184	31	0.86 (0.63-1.16)	0.309	0.80 (0.45-1.43)	0.457	0.039

AOR: adjusted odds ratio; CI: confidence interval; HWE: Hardy–Weinberg equilibrium. ^a^Adjusted for age and gender for dominant model. ^b^Adjusted for age and gender for recessive model.

**Table 2:** Stratification analysis between *METTL1* genotypes and glioma risk.

Variables	rs2291617 (cases/controls)		AOR (95% CI)^a^	*P* ^a^	rs10877012 (cases/controls)		AOR (95% CI)^a^	*P* ^a^	Risk genotypes^b^ (cases/controls)		AOR (95% CI)^a^	*P* ^a^
	GG/GT	TT			TT/TG	GG			0	1–3		
Age, month												
<60	109/153	24/21	1.60 (0.85-3.03)	0.145	111/155	22/19	1.61 (0.83-3.12)	0.156	109/153	24/21	1.60 (0.85-3.03)	0.145
≥60	147/177	32/29	1.32 (0.76-2.28)	0.323	146/177	33/29	1.37 (0.80-2.37)	0.256	145/177	34/29	1.42 (0.83-2.44)	0.205
Gender												
Females	119/140	26/24	1.23 (0.67-2.26)	0.513	119/141	26/23	1.29 (0.70-2.39)	0.420	118/140	27/24	1.29 (0.70-2.36)	0.417
Males	137/190	30/26	1.61 (0.91-2.85)	0.102	138/191	29/25	1.60 (0.90-2.85)	0.113	136/190	31/26	1.67 (0.95-2.94)	0.076
Subtypes												
Astrocytic tumors	173/330	39/50	1.47 (0.93-2.35)	0.101	173/332	39/48	1.53 (0.96-2.44)	0.076	171/330	41/50	1.56 (0.99-2.47)	0.057
Ependymoma	51/330	10/50	1.42 (0.66-3.03)	0.366	52/332	9/48	1.35 (0.61-2.96)	0.461	51/330	10/50	1.42 (0.66-3.03)	0.366
Neuronal and mixed	20/330	5/50	1.68 (0.60-4.70)	0.325	20/332	5/48	1.75 (0.62-4.92)	0.287	20/330	5/50	1.68 (0.60-4.70)	0.325
Embryonal tumors	10/330	2/50	0.93 (0.19-4.71)	0.934	10/332	2/48	0.95 (0.19-4.83)	0.954	10/330	2/50	0.93 (0.19-4.71)	0.934
Clinical stages												
I + II	183/330	41/50	1.46 (0.93-2.29)	0.103	186/332	38/48	1.39 (0.87-2.20)	0.169	183/330	41/50	1.46 (0.93-2.29)	0.103
III + IV	72/330	15/50	1.32 (0.70-2.50)	0.388	70/332	17/48	1.61 (0.87-2.97)	0.131	70/330	17/50	1.54 (0.83-2.84)	0.168

AOR: adjusted odds ratio; CI: confidence interval. ^a^Adjusted for age and gender, omitting the corresponding stratify factor. ^b^Risk genotypes were carriers with rs2291617 TT, rs10877013 CC, and rs10877012 GG genotypes.

**Table 3:** Stratification analysis between *WDR4* genotypes and glioma risk.

Variables	rs15736 (cases/controls)		AOR (95% CI)^a^	*P* ^a^	Protective genotypes^b^ (cases/controls)		AOR (95% CI)^a^	*P* ^a^
	GG	GA/AA			0–4	5		
Age, month								
<60	112/138	21/36	0.71 (0.39-1.29)	0.256	112/139	21/35	0.73 (0.40-1.34)	0.312
≥60	154/161	25/45	0.58 (0.34-0.995)	0.048	154/162	25/44	0.60 (0.35-1.02)	0.061
Gender								
Females	123/123	22/41	0.55 (0.31-0.98)	0.042	123/124	22/40	0.57 (0.32-1.01)	0.055
Males	143/176	24/40	0.72 (0.41-1.25)	0.238	143/177	24/39	0.74 (0.42-1.29)	0.291
Subtypes								
Astrocytic tumors	184/299	28/81	0.54 (0.34-0.87)	0.011	184/301	28/79	0.60 (0.35-0.90)	0.016
Ependymoma	50/299	11/81	0.82 (0.40-1.67)	0.575	50/301	11/79	0.84 (0.41-1.71)	0.620
Neuronal and mixed	21/299	4/81	0.73 (0.24-2.19)	0.572	21/301	4/79	0.75 (0.25-2.27)	0.614
Embryonal tumors	10/299	2/81	0.72 (0.15-3.40)	0.673	10/301	2/79	0.74 (0.16-3.53)	0.706
Clinical stages								
I + II	193/299	31/81	0.58 (0.37-0.92)	0.019	193/301	31/79	0.60 (0.38-0.95)	0.029
III + IV	73/299	14/81	0.70 (0.37-1.30)	0.257	73/301	14/79	0.72 (0.39-1.35)	0.307

AOR: adjusted odds ratio; CI: confidence interval. ^a^Adjusted for age and gender, omitting the corresponding stratify factor. ^b^Protective genotypes were carriers with rs2156315 CC/CT, rs2156316 CG/GG, rs6586250 CT/TT, rs15736 GA/AA, and rs2248490 CG/GG genotypes.

## Data Availability

All the data were available upon request.

## Abbreviations

CNS: Central nervous system
m^7^G: N7-methylguanosine
eQTL: Expression quantitative trait loci
CBTRUS: Central Brain Tumor Registry of the United States
LGG: Low-grade glioma
WHO: World Health Organization
pHGG: Pediatric high-grade glioma
GWAS: Genome-wide association study
SNP: Single nucleotide polymorphism
WDR4: WD repeat domain 4
METTL1: Methyltransferase-like 1
HWE: Hardy-Weinberg equilibrium
OR: Odds ratio
CI: Confidence interval
GTEx: Genotype-tissue expression
UTR: Untranslated region
ESCC: Esophageal squamous cell carcinoma
ICC: Intrahepatic cholangiocarcinoma
HCC: Hepatocellular carcinoma
EGFR: Epidermal growth factor receptor
pri-miRNA: Primary miRNA
mESC: Mouse embryonic stem cell.

## Supplementary Materials

Supplementary Materials Table S1: frequency distribution of selected variables in glioma patients and cancer-free controls in combined subjects.
